# The Role of Temperature in Shaping Mosquito-Borne Viruses Transmission

**DOI:** 10.3389/fmicb.2020.584846

**Published:** 2020-09-25

**Authors:** Rachel Bellone, Anna-Bella Failloux

**Affiliations:** ^1^Department of Virology, Arboviruses and Insect Vectors, Institut Pasteur, Paris, France; ^2^Sorbonne Université, Collège Doctoral, Paris, France

**Keywords:** temperature, arboviruses, vector-borne diseases, mosquitoes, vectorial capacity

## Abstract

Mosquito-borne diseases having the greatest impact on human health are typically prevalent in the tropical belt of the world. However, these diseases are conquering temperate regions, raising the question of the role of temperature on their dynamics and expansion. Temperature is one of the most significant abiotic factors affecting, in many ways, insect vectors and the pathogens they transmit. Here, we debate the veracity of this claim by synthesizing current knowledge on the effects of temperature on arboviruses and their vectors, as well as the outcome of their interactions.

## Introduction

**FIGURE 1:**
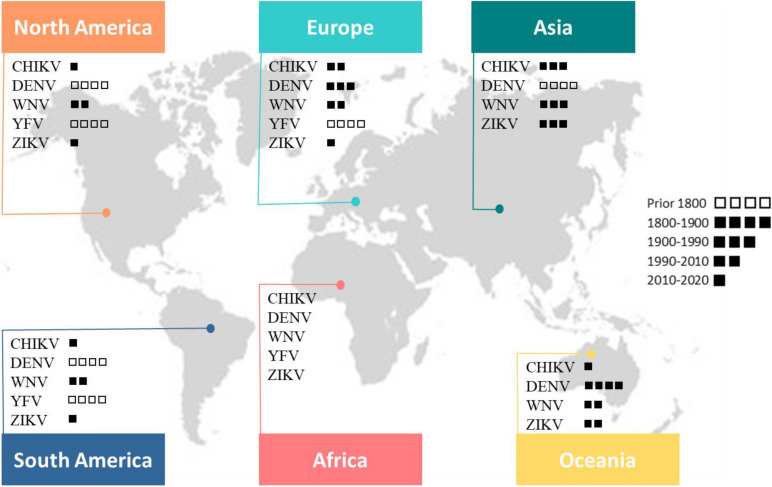
This map shows the global distribution of five arboviruses (current or past local transmission). Little squares refer to the period of first documented detection in humans (virus introduction). Phylogenetic studies suggest an African origin for all five viruses (Braack et al., 2018).

Viral pathogens with high epidemic potential have been historically a major concern for global economies and health. Over the past decades, efforts put into vaccination programs, alongside the development of effective antiviral treatments, have led to major medical advances. However, in a constantly changing world, viral diseases remain a major challenge; infectious agents continuously evolve and find opportunities to emerge (Malik Peiris and Parrish, 2011). Over time, vector-borne diseases (VBDs) have become increasingly important, reaching nearly 30% of emerging infectious disease events (Jones et al., 2008). More precisely, in the past 30 years, mosquito-borne viruses (MBVs) have dramatically expanded their distribution range within increasingly frequent and large epidemics (Gubler, 1996, 2002b; Mayer et al., 2017). MBVs such as Zika virus (ZIKV; *Flaviviridae*, *Flavivirus*), dengue virus (DENV; *Flaviviridae*, *Flavivirus*), yellow fever virus (YFV; *Flaviviridae*, *Flavivirus*), West Nile virus (WNV; *Flaviviridae*, *Flavivirus*), and chikungunya virus (CHIKV; *Togaviridae*, *Alphavirus*) have been responsible for millions of human cases with significant morbidity and mortality over the last decade (Gould et al., 2017; Figure 1).

In 2016, ZIKV was designated as a public health emergency of international concern by the World Health Organization (WHO). Indeed, 50 years after its first isolation from a human case in East Africa (Simpson, 1964; Gubler et al., 2017), ZIKV largely spread in the Pacific islands and the Americas accompanied by unusual notifications of microcephaly in newborns. The sudden and rapid spread of ZIKV received widespread media coverage, yet the upsurge or expansion of historical flaviviruses (YFV and DENV) is also of major importance and raises questions about our ability to eliminate arboviruses. DENV is one of the most widespread MBV with nearly half of the world’s population (128 countries) at risk of infection (Brady et al., 2012; Messina et al., 2014); it affects annually 50–100 million people (Bhatt et al., 2013) at a cost of US$ 8–9 billion (Shepard et al., 2016). While classic dengue fever occurs practically everywhere in the distribution range of its principal vector, *Aedes aegypti*, dengue hemorrhagic fever is more widespread in South-East Asia and tropical America. In the absence of vaccines without restriction use and specific antiviral treatments, the dengue situation is worsening with a notable rise in mortality rates (Ong et al., 2007). In addition, YFV originated from Africa, caused devastating urban epidemics from the 18th to the early 20th century in the Americas (Monath and Vasconcelos, 2015). The development of vaccines in the 1930s as well as mosquito control programs led to an elimination of urban yellow fever. While YFV infections are now mainly acquired in the forest cycle, the number of urban human cases is increasing, evidencing a significant epidemiological change of yellow fever in South America (Vasconcelos and Monath, 2016). Another important member of the *Flavivirus* genus, WNV, transmitted by *Culex* species mosquitoes, gradually became the most widely distributed MBV (Chancey et al., 2015). In the late 1990s, the epidemiology and clinical spectrum of WNV remarkably changed. Notably, in 1999, from a small focus in New York City, WNV spread throughout the United States (US) accompanied by the detection of encephalitis cases. In the following years, the virus spread throughout the continent and in 2002, the United States recorded the largest outbreak of West Nile meningo-encephalitis ever documented in the world (Sejvar, 2003). The WNV outbreak in the United States (1999–2010) is a reminder that the importation and establishment of vector-borne pathogens outside their original distribution range represents a serious threat to the world^1^. Following the WNV episode, the American continent was again hit by another MBV in 2013; this time, it was an *Alphavirus*, the CHIKV. After decades of sporadic outbreaks in Africa and Asia, CHIKV finally emerged as a global pathogen causing large scale epidemics in Africa, Asia, America and to a lesser extent, in Europe (Zouache and Failloux, 2015; Silva and Dermody, 2017). CHIKV caused the first local *Aedes albopictus*-vectored virus transmission in Europe, with hundreds of cases in Italy and France (Grandadam et al., 2011; Calba et al., 2017; Rezza, 2018), less than 20 years after the first detection of *Ae. albopictus* in Italy (Sabatini et al., 1990). Later, *Ae. albopictus* was responsible for local cases of DENV and ZIKV in Croatia, France, and Spain (Gjenero-Margan et al., 2011; Aranda et al., 2018; Brady and Hay, 2019). The changing epidemiology of arboviral diseases results from a complex set of factors (Gubler, 2002a; Gould et al., 2017), combining both intrinsic and extrinsic interactions (Lambrechts et al., 2009; Zouache et al., 2014; Gloria-Soria et al., 2017).

In addition to human activities and globalization as key contributors in vector and pathogen spread, climate change exerts determinant effects on VBDs (Reeves et al., 1994; Gould and Higgs, 2009; Mills et al., 2010; IPCC, 2014). Because arthropods are poikilothermic ectotherms (i.e., body internal temperature is not constant and depends on temperature of surrounding environment), they are highly vulnerable to temperature changes, as are the pathogens they host. Environmental temperature is then, one of the most important abiotic factors influencing mosquito ecology, behavior, physiology and ultimately, virus transmission (Samuel et al., 2016; Reinhold et al., 2018). This review compiles the current knowledge on the effects of temperature on mosquito and virus biology with the goal of understanding how collectively these effects have an impact on virus-mosquito interactions and viral transmission.

## Main Features of Mosquito-Borne Diseases

### Clinical Aspects

Most mosquito-borne viral infections are asymptomatic or non-specific mild infections (Endy et al., 2011). Symptomatic infections are often identified as a systemic febrile illness with non-specific symptoms, thus making mosquito-borne diseases (MBDs) very difficult to diagnose (Pezzi et al., 2019). Headache, weakness and rashes are common manifestations. Muscle and joint aches are also very frequent, especially with *Alphavirus* infections, for which these symptoms can persist for months or even years. In most cases, MBDs febrile illness ends with a full recovery. In only a few cases, illness may evolve to more severe forms, which principally include hepato-nephritis, hemorrhagic fever, and encephalitis (Young, 2018). Currently, there are only two marketed vaccines [against YFV and Japanese encephalitis virus (JEV)] and treatments are not specific. It is worth mentioning that asymptomatic cases are epidemiologically important, although clinically unapparent. In absence of symptoms, people can still transmit the virus either because they have sufficient viremia to participate to the natural transmission cycle (Duong et al., 2015) or because of other transmission routes such as blood transfusion or organ transplantation (Chastel, 2011).

### Complex Transmission Cycles

Mosquito-borne viruses circulate primarily within enzootic cycles between zoophilic mosquitoes and wild animals. Spillovers of enzootic viruses from a sylvatic cycle occur when anthropo-zoophilic mosquitoes serve as bridge vectors for transmission of the virus from animals to humans. It is then, an opportunity for the virus to enter epidemic cycles where mosquitoes are vectors and humans are either (i) dead-end host, because infection does not lead to a viremia high enough to infect mosquitoes [e.g., West Nile virus (WNV), Venezuelan equine encephalitis virus (VEEV) or JEV] or (ii) reservoir and amplification host, when infection in humans leads to high viremia, ensuring an inter-human transmission (e.g., DENV, ZIKV, CHIKV, and YFV) (Young, 2018; Valentine et al., 2019). In the first scheme, epidemics rely on regular virus spillover from enzootic cycles while in the second scheme, epidemic cycles are self-sustaining, having lost the requirement of enzootic cycles to cause outbreaks (Weaver and Reisen, 2010). Some transmission cycles may be relatively simple with a main vector and a main host species (e.g., DENV, CHIKV, and ZIKV), while some others are more complex, involving several host and vector species [e.g., Rift Valley Fever Virus (RVFV), JEV, WNV] (Young, 2018).

## Virus Cycle in the Vector and Effects of Temperature

Mosquito-borne viruses are arthropod-borne viruses (arboviruses), a group of viruses typically transmitted from infected to susceptible vertebrate hosts by hematophagous arthropods (vectors). Most arboviruses causing human diseases belong to three main families: *Flaviviridae* (genus *Flavivirus*), *Togaviridae* (genus *Alphavirus*) and *Phenuiviridae* (genus *Phlebovirus*). These viruses share different types of genomes, structural organization and replication strategies (Holmes, 2003; Duffy et al., 2008; Bradwell et al., 2013), but have in common, single stranded RNAs as carriers of their genetic information.

### Characteristics of RNA Genomes

Because of their chemical structures, RNAs appears as an unreliable support for transmission of genetic information in comparison to DNA (Lindahl, 1996; Lazcano et al., 1988; Krokan et al., 2002). A striking attribute of RNA viruses is their high mutation rate. During RNA replication, the mutational rate is in the range of 10^–6^ to 10^–4^ substitutions per nucleotide, corresponding roughly to 0.01 to 1 mutation per 10 kb of genome (Domingo and Holland, 1997; Lauring et al., 2013). This high mutation rate greatly contrasts with that of prokaryotic or eukaryotic genomes (Drake et al., 1998; Drake, 1999; Nachman and Crowell, 2000), as well as with that of DNA viruses (range of 10^–8^ to 10^–6^) (Sanjuan et al., 2010). The high fidelity of DNA replication is not merely insured by the accuracy of DNA polymerases but also by the combined action of proofreading enzymes and mismatch repair system (Sanjuan and Domingo-Calap, 2016). The absence of such correction activity during RNA replication results in a more error-prone replication mode (Steinhauer et al., 1992). Consequently, RNA viruses circulate as dynamic mutant clouds of closely related genome sequences referred to as a quasispecies (Domingo et al., 2008). In the multi-component viral system, replicative infectious particles coexist with defective genomes, including defective interfering genomes (DIs) that are degenerated and non-replicative forms of viral genomes. DIs depend on the co-infection with the self-infectious virus for replication and play a significant role in triggering immune responses, modulating disease outcome and influencing virus replication and evolution (Rezelj et al., 2018). High mutation rates and short generation times are largely responsible for the extremely high genetic variability of RNA virus populations (Moya et al., 2000). Other source of variation in RNA viruses include recombination and reassortment (Domingo and Holland, 1997). Overall, RNA virus populations behave as huge reservoirs of mutants that permit rapid adaptation to environmental changes. In the mutant cloud, sequences are subject to constant genetic changes, competition and selection for the most suitable combination of viral genomes (Andino and Domingo, 2015; Domingo and Perales, 2019). RNA viruses can then exploit multiple adaptive solutions to overcome selective pressures such as immune responses, antiviral therapies or fluctuating environments.

### Dual Host Cycling and Virus Adaptation

Despite inherent potential for mutation and subsequent adaptation, arboviruses exhibit lower mutational rates than non-vectored RNA viruses (by a factor of 10) (Jenkins et al., 2002; Vasilakis et al., 2009). This evolutionary stasis is generally attributed to the alternated transmission between hosts belonging to two disparate phyla, each of which presenting different demands for viral replication. According to the trade-off hypothesis, the virus is constrained to a compromised fitness for an optimal replication in both vector and vertebrate hosts (Coffey et al., 2008; Ciota and Kramer, 2010), although this explanation is not always well supported by other studies (Novella et al., 1999; Ciota et al., 2009; Deardorff et al., 2011).

**FIGURE 2:**
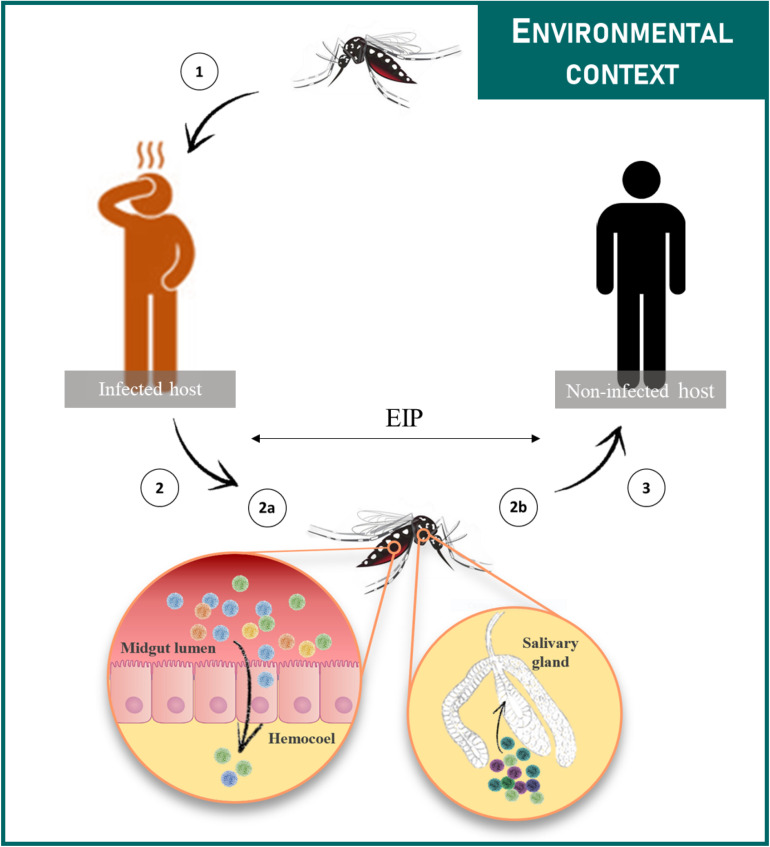
Female mosquitoes acquire the virus during a blood meal on a viremic host (1). Then the virus infects the midgut epithelium from which it escapes and disseminates to peripheral tissues/organs (2a). The virus reaches the salivary glands (2b) in which it replicates prior to be released in saliva during a blood meal (3). The time between the ingestion of the virus and its presence in saliva is referred to as the EIP. Transmission cycles are influenced by multiple extrinsic environmental factors.

Transmission occurs when the virus ingested by the vector replicates successfully in midgut epithelial cells, peripheral tissues/organs and salivary glands, prior to its expectoration through the insect bite (Figure 2). During this journey in the vector, viral populations undergo successive genetic bottlenecks that greatly modify initial population structures (Forrester et al., 2014). A study performed with DENV-2 showed that more than 90% of single nucleotide variants (SNVs) were lost upon transmission from infected patients to *Ae. aegypti* mosquitoes, as well as from mosquito midgut to salivary glands; new variants were generated at each step of infection in vector, thereby maintaining the level of viral diversity (Coffey et al., 2013; Sim et al., 2015). Bottlenecks encountered by viruses differ according to mosquito species and probably, mosquito-virus combinations (Grubaugh et al., 2016). Viruses are thus exposed to the specific selective pressures of their hosts. These selective pressures, including virus entry (appropriate receptors), microbiota and immune responses, directly shape virus evolutionary patterns and ultimately, their potential to emerge (Ruckert and Ebel, 2018). For instance, the adaptive amino acid change from alanine to valine at the position 226 of the E1 glycoprotein (E1-A226V) of the East/Central/South African (ECSA) CHIKV arose from an unusual transmission by *Ae. albopictus* instead of the classical vector *Ae. aegypti*. This specific mutation led to an enhanced transmission of CHIKV by *Ae. albopictus* (Tsetsarkin et al., 2007; Vazeille et al., 2007) causing 266 000 human cases during the 2005–2006 epidemic on La Réunion island (Schuffenecker et al., 2006). However, the benefit of the E1-A226V mutation in *Ae. albopictus* may vary according to the environmental temperature (Zouache et al., 2014). As vectors are exposed to a multitude of environmental variables, which can modify their intrinsic properties, consequences on viral populations are expected; viral populations may explore possibilities of adaptation and thus follow evolutionary processes associated with molecular and phenotypic changes (Ciota and Kramer, 2010; Coffey et al., 2013).

### Effects of Temperature on Viral Populations

In their vertebrate host, arboviruses replicate at temperatures ranging from 37°C up to 44°C (Kinney et al., 2006), then switch to their ectotherm vectors where temperatures vary depending on the ambient temperature (as low as ∼15°C; Table 1). The fact that arboviruses tolerate such drastic temperature changes raises a number of questions: the effects of temperature on quasispecies structures and dynamics, the selection of temperature-adapted variants and impacts on virus transmission, expansion and pathogenesis. Temperature is known to induce molecular changes that impact lipids, nucleic acids and protein structures and functions (Pain, 1987). Temperature is thus very likely to modify properties of virions and their interactions with cellular components during replication. Studies on enzyme functional attributes most sensitive to temperature indicate that ligand binding affinity and catalytic rate are key targets during temperature adaptation; ligand affinity decreases during cold adaptation to allow more rapid catalysis (Fields et al., 2015). Whereas, thermodynamic models suggest that at low temperatures, enhanced cell binding may compensate for lower replication kinetic rates such that some transmission can still occur (Gale, 2019). Some DENV serotypes undergo a temperature dependent conformational change from a “smooth” form at lower temperatures to a “bumpy” form at temperatures approaching 37°C (Rey, 2013; Zhang et al., 2015). In addition, alphaviruses were found to enter mammalian cells *via* a temperature-dependent alternative mechanism. At temperatures that inhibit virus receptor-mediated endocytosis (classical entry for arboviruses), viral genomes were internalized directly at the plasma membrane (Vancini et al., 2013). Furthermore, silent mutations (i.e., mutations without amino acid sequence changes) are believed to play important roles in adaptation of RNA virus to elevated temperature (Kashiwagi et al., 2014). Temperature could influence the incidence and severity risk of arboviruses outbreaks by altering arboviruses evolution, selection and transmission.

**TABLE 1:** Effect of temperature on vector competence.

**Virus strains**	**Mosquito Species**	**Mosquito populations**	**Temperatures**	**Results**	**References**
**DENV**					
DENV-2 (New Guinea C)	*Ae. albopictus*	China (Foshan)	18, 23, 28, and 32°C	EIP was shorter at the highest temperature	Liu et al., 2017
DENV-2 (New Guinea C)	*Ae. albopictus*	China (Shangai)	18, 21, 26, 31, and 36°C	EIP gradually decreased when temperature increased. Infection rates increased along with temperature until 31°C	Xiao et al., 2014
DENV-2 (Kenya, 2012)	*Ae. aegypti*	Kenya (Kilifi and Nairobi)	26 and 30°C	Infection rates were higher at 30°C	Chepkorir et al., 2014
DENV-2 (434S and 6H)	*Ae. aegypti*	Vietnam (Hanoi and Ho Chi Minh)	25, 27, and 32°C	The mosquito population from Ho Chi Minh City was more susceptible to infection at lower temperature than the mosquito population from Hanoi. For both virus strains, highest infection rates were obtained at 25°C for the mosquito population from Ho Chi Minh City	Gloria-Soria et al., 2017
**ZIKV**					
Asian lineage (PRVABC59)	*Ae. aegypti*	United States (California)	18, 21, 26, and 30°C	EIP decreased as temperature increased. At 18°C, a 15% transmission efficiency was reached at day 31 post infection (pi), while a 100% transmission was observed at 21 days pi at 30°C	Winokur et al., 2020
Asian lineage (FB-GWUH-2016)	*Ae. japonicus*	Germany	21, 24, and 27°C	Infection rates increased with temperature and virus transmission was detected exclusively at 27°C	Jansen et al., 2018
**WNV**					
WN-FL03p2–3	*Cx. quinquefasciatus*	United States (Florida)	25, 28, and 30°C	Infection rates increased as temperature increased	Richards et al., 2007
NY99_crow 397-99	*Cx. pipiens*	United States (New-York)	18, 20, 26, and 30°C	Infection rates increased as temperature increased	Dohm et al., 2002
NY99-3356 and WN02-1956	*Cx. pipiens*	United States (Pennsylvania)	15, 18, 22, and 32°C	EIP was significantly shorter and transmission rates higher at 32°C	Kilpatrick et al., 2008
WNV lineage 2 (Greece 2010)	*Cx. pipiens*	Netherlands (Amsterdam and Best)	18, 23. and 28°C	Biotype *pipiens* and hybrids showed significant increased transmission rates at higher temperatures. Biotype *molestus* transmission rate did not increase with temperature	Vogels et al., 2016
**CHIKV**					
ECSA; (Mauritius, 2006) Asian; (Caribbean, 2014)	*Ae. albopictus*	Australia	18 and 28°C	EIP was shorter at 28°C. At 18°C, mosquitoes infected with the Asian genotype showed no evidence of virus in saliva even at the latest analysis time point (7 days post infection)	Wimalasiri-Yapa et al., 2019
ECSA (CNR_24/2014)	*Ae. albopictus*	Germany, Italy	18, 21, and 24°C	Transmission rates were higher at lower temperatures	Heitmann et al., 2018
ECSA (Lamu001)	*Ae. aegypti*	Kenya (Western and Coastal regions)	26 and 32°C	Western mosquitoes exhibited higher infection rates at 32°C than at 26°C. In contrast, coastal mosquitoes did not show any statistical difference in infection rates whatever the temperature	Mbaika et al., 2016

## Mosquito Biology, Physiology, Behavior and Effects of Temperature

Mosquitoes are the principal vector of arboviruses, although other arthropod taxa such as ticks, sandflies and biting midges are also implicated in medically important arboviruses transmission (Alkan et al., 2013; Kazimirova et al., 2017; Sick et al., 2019). Mosquitoes have a complex life cycle characterized by complete metamorphosis and four distinct life stages: egg, larva, pupa and adult. The first three stages occur in water whereas adults are active flying insects, with females being hematophagous. The period between blood feeding and eggs laying corresponds to the gonotrophic cycle; females undergo successive gonotrophic cycles during their entire life.

### Effects of Temperature on Mosquito Distribution and Life History Traits

Environmental temperature is an overriding factor defining the geographic distribution range limits of many organisms, in particular ectotherms. Mosquitoes can only survive and reproduce in suitable environments that depend on the ecological characteristics of the mosquito species. Many studies have used temperature to map global or regional suitability and distribution of mosquito species (Nawrocki and Hawley, 1987; Brady et al., 2013, 2014; Samy et al., 2016; Ding et al., 2018). With their model, Kraemer et al. (2015) estimated *Ae. aegypti* and *Ae. albopictus* distributions to be now extensive in all continents, including North America and Europe. For both species, the most important predictor of distribution was temperature (Kraemer et al., 2015). Sensitivity of mosquitoes to temperature reflects the effects of temperature on the main mosquito physiological processes.

First, temperature has a significant effect on eggs viability and hatching time, with optimal temperatures for hatching depending on mosquito species (Hawley et al., 1989; Hanson and Craig, 1994; Impoinvil et al., 2007; Mohammed and Chadee, 2011). Eggs of the temperate mosquito, *Ae. albopictus*, tolerate lower temperatures than eggs of tropical *Ae. aegypti* (Hanson and Craig, 1994); this contributes significantly to the larger geographical distribution of *Ae. albopictus* encompassing both tropical and temperate regions (Kraemer et al., 2015). Besides, eggs of *Ae. albopictus* from temperate regions have lower lethal temperatures than eggs of the same species from tropical regions (Hanson and Craig, 1995), emphasizing thermal acclimation. Along with photoperiod, cold temperatures trigger the production of diapausing eggs in *Ae. albopictus*. Diapause is a genetically programmed mechanism crucial for mosquitoes to overwinter (Thomas et al., 2012); it is a hormonally controlled developmental arrest that is triggered by unfavorable environmental conditions and lifted with the return of favorable conditions (Denlinger and Armbruster, 2014).

Secondly, larvae and pupae are strictly aquatic stages and thus submitted to temperature variations of breeding sites. In addition to other factors such as nutrient availability, competition for food, presence of predators/parasites, pollution with organic matter and chemicals, temperature is critical for the survival, development and emergence time of immature stages (Tun-Lin et al., 2000; Bayoh and Lindsay, 2004; Delatte et al., 2009; Dodson et al., 2012). Notably, survival of immature stages was compromised below 16°C and above 38°C for *Ae. aegypti* with an optimal survival rate at 26°C (Carrington et al., 2013). It is commonly found that as temperature increases, immature stages development time decreases (Shelton, 1973; Loetti et al., 2011; Dodson et al., 2012; Grech et al., 2015), up to a critical thermal threshold for survival (Delatte et al., 2009; Loetti et al., 2011). Development time of *Ae. aegypti* (from egg to adult) was inversely proportional to temperature, ranging from 7.2 days at 35°C to 39.7 days at 15°C (Tun-Lin et al., 2000). In addition, temperature stress during immature stages have carry-over effects on adult life traits such as fecundity, survival (Ezeakacha and Yee, 2019) and body size, with cooler temperatures producing larger mosquitoes (Mohammed and Chadee, 2011; Dodson et al., 2012). Correlations between body size and other physiological features such as blood feeding behavior have been largely discussed (Xue et al., 1995; Scott et al., 2000; Farjana and Tuno, 2013). Small mosquitoes presented exacerbated host-seeking behavior with multiple attempts to blood feed, enhancing contact frequency with hosts (Farjana and Tuno, 2013) while conflicting results were obtain elsewhere (Xue et al., 1995).

Regardless of temperature stress encountered during immature stages, temperature also has a direct impact on mosquito adult stage. In *Culex* species, female longevity significantly increased when adult holding temperature decreased (Ciota et al., 2014). Similarly, *Ae. albopictus* adult survival was inversely correlated with temperature, presenting the highest survival rate at 15°C and the lowest at 35°C (Delatte et al., 2009). Mosquito flight activity is important for many life history traits such as reproduction, nutrition and host-seeking. *Ae. aegypti* is able to fly in a temperature range of 15 to 32°C with an optimum at 21°C (Rowley and Graham, 1968). The gonotrophic cycle length is an indicator of mosquito abundance and a proxy of contact frequencies between hosts and vectors. The shorter the gonotrophic cycle, the more often females will come in contact with their hosts and the more generations will be produced, resulting in higher mosquito densities and greater population genetic diversity (increased potential to adapt to contrasting environments). In *Ae. aegypti*, the duration of the gonotrophic cycle decreases when temperature increases. Temperature also influences the time for the first blood meal; females kept at higher temperatures have their first blood meal within 48 h of emergence. Finally, temperature had a significant effect on the number of eggs laid that peaks at nearly 80 eggs per female when reared at 26°C (Carrington et al., 2013).

It is noteworthy to mention that results obtained under constant temperatures can vary from those under fluctuating temperatures that better mimic field conditions. Temperature effects on life history traits like adult reproduction, larval survival and development time depend on the combination of mean temperature and magnitude of fluctuations (Lambrechts et al., 2011; Carrington et al., 2013; Liu-Helmersson et al., 2014).

### Effects of Temperature on Microbiota

Mosquitoes harbor very complex, abundant and dynamic microbial communities that are found at high concentrations in the different intestinal portions (Dillon and Dillon, 2004; Engel and Moran, 2013). These micro-organisms are comprised of protozoans, fungi, bacteria and viruses (Guegan et al., 2018b; Strand, 2018). The combination of the host and its associated microbiota is referred to as the holobiont. This concept, where macro-organisms and their microbiota are seen as a cooperative unit, highlights the strong symbiotic interactions within the holobiont. Most of the literature focuses on bacterial communities, which were shown to play a major role in insect biology (contribution to nutrition, protection against pathogens, modulation of immune responses) (Engel and Moran, 2013; Guegan et al., 2018a; Sicard et al., 2019). While mosquitoes naturally host multiple bacteria [genera *Acinetobacter*, *Asaia*, *Delftia*, *Pseudomonas*, *Wolbachia*, *Bacillus* (Zouache et al., 2011)], their role in priming the mosquito immune responses is largely debated; the mosquito’s microbiota elicits basal immune responses which act against pathogens and reduce density of the midgut microbial load itself (Ramirez et al., 2012). Temperature is a substantial factor shaping microbial communities of organisms, especially in insects (Prado et al., 2010; Lokmer and Mathias Wegner, 2015; Kikuchi et al., 2016; Moghadam et al., 2018). It was shown in flies that developmental temperature is a decisive factor influencing bacterial community structures with consequences on the flies’ thermal tolerance (Moghadam et al., 2018). Similarly, in mosquitoes, temperature may affect midgut microbial diversity and structure. Compositional changes in *Ae. albopictus* midgut microbiota were found to be induced by a temperature decrease (Guegan et al., 2018a). Furthermore, higher temperatures were shown to reduce *Wolbachia* abundance in *Culex pipiens/restuans* mosquitoes, which in turn, was correlated with a higher susceptibility to WNV in subsequent mosquito generations and higher prevalence of the virus (Novakova et al., 2017).

### Effects of Temperature on Mosquito Gene Expression and Immunity

Regulation of gene expression is a common mechanism that organisms use to adapt their phenotypes and maintain fitness in response to stressors such as temperature (Pigllucci, 1996; Morris and Rogers, 2014). *Ae. aegypti* adult mosquitoes held at 20°C had a very different transcriptomics profile from those held at 28°C, whereas at a higher temperature (36°C), mosquitoes showed no significant transcriptional differences from the standard holding temperature of 28°C (Gonçalves Ferreira et al., 2020). Genes, whose expression were altered at 20°C, are involved in various aspects of mosquito biology: blood-meal digestion, ROS metabolism and mosquito innate immunity (Gonçalves Ferreira et al., 2020). Mosquito immunity constitutes a powerful protection in symbiotic, entomopathogenic and mosquito-borne pathogen infections. To defend against pathogens, mosquitoes activate several immune-signaling pathways: Toll, JAK-STAT, Imd/JNK and RNAi (RNA interference) pathways (Fragkoudis et al., 2009; Kumar et al., 2018). Temperature has complex effects on mosquito immune functions (Murdock et al., 2012). In *Ae. aegypti*, a differential expression of immune-specific and detoxification genes was found in fourth instar larvae exposed to temperature stress (32°C). Thermal stress undergone during larval development had also remnant effects on adults’ genes expression levels (Muturi et al., 2011). RNAi is the most significant antiviral immune response in mosquitoes (Blair, 2011). The triggering of the RNAi pathways can be destabilized when mosquitoes are reared at cooler temperatures (18°C) (Adelman et al., 2013). Impairment of immune barriers may profoundly affect mosquito interactions with symbionts, entomopathogenic organisms and mosquito-borne pathogens but also interactions of these latter together. Studies have correlated the alteration of the immune system by thermal stress with changed susceptibility to CHIKV, YFV, and Sindbis virus (SINV) (Muturi et al., 2011; Adelman et al., 2013). The subtle balance between effects of temperatures on mosquito antiviral immune responses and virus replication likely conditions the outcome of virus transmission.

Collectively, by affecting mosquito biology, physiology and behavior, temperature plays a key role in mosquito dynamics (El Moustaid and Johnson, 2019). Changes in mosquito geographical distribution, survival, development time, gonotrophic cycle, microbiota or immune responses can have critical impacts on their role as vector of pathogens (Waldock et al., 2013a; da Cruz Ferreira et al., 2017).

## Temperature and Its Potential to Influence Arboviruses Transmission

### Major Concepts in Medical Entomology

**FIGURE 3:**
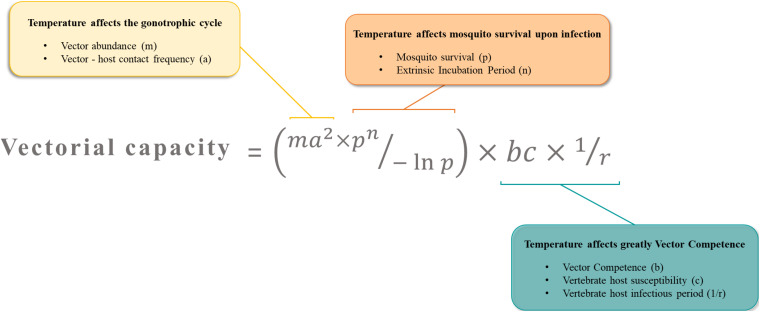
The vectorial capacity represents the number of potential infectious bites that a vector dispenses after the EIP is completed. It describes the efficiency at which a vector population transmits a pathogen in natural settings. Temperature is a key factor affecting major parameters in this equation.

Arboviruses emergence is driven by the need of their arthropod vectors to uptake blood from vertebrate hosts. During a blood meal on a viremic host, female mosquitoes ingest the virus along with the blood. To transmit the virus to following hosts, the mosquito has to be competent for the virus (Figure 2). A key concept in medical entomology, vector competence is defined as the ability of an arthropod vector to uptake and transmit afterward a given pathogen (Hardy et al., 1983). In mosquitoes, it is generally determined by the crossing of four major barriers: (Malik Peiris and Parrish, 2011) the midgut infection barrier, (Jones et al., 2008) the midgut escape barrier, (Gubler, 1996) the salivary gland infection barrier and (Gubler, 2002b) the salivary gland escape barrier (Franz et al., 2015). Basically, the virus infects and replicates in the midgut epithelium before crossing the midgut basal lamina and disseminates to peripheral tissues/organs. Ultimately, the virus reaches the salivary glands in which it replicates prior to be released in the saliva (Figure 2). Competent females develop persistent infections, thus transmitting the virus for the rest of their lives at each blood meal on susceptible hosts *via* the injection of infectious saliva. Vector competence is controlled by both intrinsic (i.e., microbiota, mosquito and virus genetic) (Hardy et al., 1983; Lambrechts et al., 2009; Jupatanakul et al., 2014; Hegde et al., 2015; Houe et al., 2019) and extrinsic (i.e., temperature) factors (Zouache et al., 2014; Ciota et al., 2018). The concept of vector competence is integrated in a broader one that is vectorial capacity which is an epidemiological measure of the transmissibility of an infectious agent by a particular vector species/population in the field (Macdonald, 1957). The mathematical equation used to evaluate the vectorial capacity (Figure 3) includes the following parameters: vector-host ratio (m), mosquito biting rate on human (a), daily survival rate (p), infectiousness of the mosquito to the vertebrate host (b), susceptibility of the vertebrate host to the virus (c), extrinsic incubation period (n) and vertebrate host infectious period (1/r) (Fontenille and Powell, 2020). Factors influencing the vectorial capacity have been largely discussed (Kramer and Ciota, 2015); the most determining variables are mosquito survival rate and extrinsic incubation period (EIP). The EIP equals the time required between the ingestion of a virus and the ability of the vector to transmit it. When this period increases, the vectorial capacity decreases. All else being equal, a virus that takes 3 days to be transmitted versus one that takes 10 days within the same vector will have a much higher epidemic potential. Indeed, the shorter the EIP, the more likely the virus is transmitted before the vector dies.

### Effects of Temperature on Vectorial Capacity

Temperature can affect important parameters of the vectorial capacity (Figure 3). First, the mosquito density, which relies on temperature-sensitive life-history traits like reproduction, gonotrophic cycles and developmental time, determines contact rates between hosts and vectors. For example, in a location where mosquitoes are at high densities, contacts between mosquitoes and hosts are increased, and so is pathogen transmission risk (Honorio et al., 2009). Second, the biting rate includes mosquito trophic preferences and requires an active host-seeking behavior which includes temperature-sensitive parameters (flight activity, gonotrophic cycle). Highly anthropophilic and active mosquitoes are more prone to transmit human pathogens and ensure their propagation. Third, mosquito survival, often negatively correlated with temperature, increases the time during which infected females serve as vectors (Christofferson and Mores, 2016). Older females are often considered as more epidemiologically dangerous, as they are more likely to have been infected and to subsequently, transmit the virus during their lifetime. Lastly, vector competence and EIP result from a complex combination of many intrinsic factors such as virus dynamics, virus and vector genetic, vector physiological traits, microbiota and immunity. Vector competence and EIP depends strongly and unimodally on temperature. Thermal optima and limits of transmission vary across vector-pathogen systems (Mordecai et al., 2019). By affecting major components of vectorial capacity, temperature may tip the scales toward either an increase or a decrease of arbovirus transmission potential.

### Principal Temperature-Dependent Factors Influencing MBV Transmission

First described as temperature dependent by Davis in 1932 (Davis, 1932), EIP is often used as an index of vector competence. Higher temperatures are associated with reduced EIP and enhanced vector competence in many virus-vector pairings (Chamberlain and Sudia, 1955; Richards et al., 2007; Xiao et al., 2014; Liu et al., 2017; Wimalasiri-Yapa et al., 2019; Winokur et al., 2020). Virus replication rates generally increase with temperature, and since dissemination and transmission correlate with viral load (Kramer and Ciota, 2015), lower temperature is generally less advantageous for arbovirus transmission. However, some exceptions sprinkle the literature; *Cx. tarsalis* mosquitoes, for example, were less competent for Western Equine Encephalitis Virus (WEEV) at 32°C than at lower temperatures (18 and 25°C) (Kramer et al., 1983). Likewise, higher infection and transmission rates were detected at lower temperature in *Ae. aegypti* infected with DENV (Heitmann et al., 2018) and in *Ae. albopictus* infected with CHIKV (Heitmann et al., 2018), respectively. These conflicting evidences are likely to result from disparate effects of temperature on virus replication and vector immune responses in each virus-vector combination.

Thermal exposure undergone during larval development has remnant effects on mosquito susceptibility to virus infection (Turell, 1993; Alto and Bettinardi, 2013). Immature stages of *Ae. albopictus* reared at lower temperatures gave adults with decreased viral dissemination when orally infected with DENV-1 (Alto and Bettinardi, 2013). Surprisingly, conflicting results were found with CHIKV and DENV-2, where susceptibility to infection increased at lower rearing temperature (Westbrook et al., 2010; Evans et al., 2018). The same trend was observed for VEEV and RVFV in *Ae. taeniorhynchus* (Turell, 1993). Interestingly, larval rearing temperature did not show any effect on *Culex* mosquito susceptibility to RVFV or WNV (Brubaker and Turell, 1998; Dodson et al., 2012). These inconsistencies may reflect different interactions between virus and vectors and may suggest the presence of interfering factors sensitive to temperature.

Several life-history traits such as adult body size vary according to rearing temperature of immature stages. Large females absorb twice the volume of blood than smaller females (Briegel, 1990), thus potentially increasing viral load ingested by larger females. *Ae. albopictus* immature stages exposed to cooler temperatures grew into larger adults with increased CHIKV susceptibility (Westbrook et al., 2010). However, smaller-sized *Ae. aegypti* and *Ae. albopictus* were significantly more likely to become infected and to disseminate DENV-2 than larger individuals (Alto et al., 2008). Indeed, smaller mosquitoes might have higher ratios of infective dose/body weight and thus acquire higher concentrations of virus per body weight. Moreover, temperature can interfere with other stressors of the larval environment. At a low temperature, high larval density led to high infection rates of adults with SINV while the reverse was observed at high temperature (Muturi et al., 2012). In addition, cooler rearing temperature of immature stages resulted in enhanced *trans-*stadial transmission (from larvae to adult) of Saint Louis encephalitis virus (SLEV) in *Aedes epactius* (Hardy et al., 1980); the number of newly emerged adults harboring the virus was significantly higher in mosquitoes reared during immature stages at 18°C than those reared at 27°C. Virus vertical transmission from females to their offspring is considered as a mechanism of persistence by which viruses are maintained in nature during unfavorable periods for horizontal transmission. Although debated, this phenomenon could have a significant impact on the subsistence and emergence of arboviruses outbreaks (Lequime and Lambrechts, 2014; Agarwal et al., 2017).

In nature, mosquitoes are submitted to daily and seasonal fluctuations of temperature. Understanding how temperature variations affect arbovirus transmission dynamics is critical to anticipate and limit the geographic and seasonal spread of MBDs. In that sense, mathematical models are pivotal tools in demonstrating the key role of temperature in VBD transmission (Huber et al., 2018; Tesla et al., 2018; Mordecai et al., 2019). More precisely, Mordecai et al. (2017) predicted in their mechanistic model that most tropical and subtropical regions are suitable for CHIKV, ZIKV, and DENV transmission during most months of the year while transmission in temperate regions is limited to only few months in summer, reducing the probability of major epidemics in those regions (Mordecai et al., 2017). In addition, thermodynamics models show that in tropical areas (mean temperature close to 29°C), small diurnal temperature range (DTR), increases DENV transmission potential while under large DTR, it decreases. Whereas in cold temperate or extremely hot climates, DENV transmission potential increases as DTR increases (Lambrechts et al., 2011; Carrington et al., 2013; Liu-Helmersson et al., 2014). Using climate change projections based on predicted temperature and DTR, mapped models showed an increasing trend over time for DENV epidemic potential in temperate regions (Liu-Helmersson et al., 2014).

The effect of climate change, especially global warming, on infectious diseases transmission has been the topic of intense debate. A large body of literature assumes that climate change will considerably modify VBDs epidemiological patterns (Reeves et al., 1994; Gould and Higgs, 2009; Mills et al., 2010; IPCC, 2014; Ryan et al., 2019). However, due to the complex interactions involved in transmission cycles and the unpredictable nature of both vectors and virus evolution, predicting the effects of climatic and environmental changes on VBDs emergence is extremely challenging (Tabachnick, 2010). Local adaptation to temperature of parasites and vectors may modulate the effects of climate change on VBDs dynamics and distribution (Sternberg and Thomas, 2014). Moreover, since mosquitoes are mobile and may move from habitats to stay in an optimal environment, they are not fully exposed to large temperature variations (Kramer, 2016). Microclimates encountered in urban areas (subways, houses…) have often higher and more stable temperatures than outdoor environments. This fosters vector survival and allows transmission cycle to persist despite adverse meteorological conditions (Haider et al., 2017). Thus, urbanization modifies climate within cities forming warmer spots; roads (with concrete and asphalt substrates) and air pollutants store the heat during daytime and release it at night, causing a rise in temperature compared to vegetated areas outside cities (Reisen, 2010). Obviously, other environmental factors including humidity, droughts, precipitation and flood act in concert with temperature to shape MBDs emergence and dynamics (Waldock et al., 2013b).

## Discussion

Typically, prevalent in the tropical belt, MBDs are now spreading, reaching even temperate regions. Over the last few decades, major pathogen-carrying vectors, like *Ae. aegypti* and *Ae. albopictus*, have significantly expanded their global distributions. MBDs burden is expected to continue to increase, especially under anticipated climate change scenarios. This situation raises many concerns about how temperature could change the current dynamics and expansion of MBDs. In this paper, we review current knowledge on the intricate interactions that reside between temperature, viruses and their vectors, in order to understand how collectively these effects may shape transmission dynamics. Temperature is one of the most significant abiotic factors affecting, in many ways, both the vectors and the pathogens they transmit. With great variance depending on vector species, populations and viral strains, temperature influences vector survival, vector population growth, distribution and genetic structure, host contact and feeding, virus susceptibility, EIP, virus structure and replication (Agarwal et al., 2017). While it is clear that factors such as high mosquito density, biting and survival rates promote transmission, it is more challenging to connect transmission to specific genetic (virus, mosquito holobiont…) and environmental variables (temperature, humidity, pollution…). Obviously, there are some combinations of factors that promote transmission while others hinder it; emergence happens when promoting factors outweigh hindering factors. There is an evident gap of information about how temperature influences virus evolution and phenotypes. In addition, deciphering how mosquito microbiota and immune functions implicated in viral transmission respond to temperature has a great importance.

In its overall effects, temperature may have a profound impact on natural ecosystems of rural and sylvatic cycles. New serotypes or currently unknown viruses could emerge from wildlife and affect humans following ecosystem alterations related to temperature changes; forest cycles are breeding grounds of unknown viruses representing a bottomless source of pathogens threatening human health (Fontenille and Powell, 2020). Neglected arboviruses are expected to become increasingly important, with temperature as a determinant factor of emergence (Lorenz et al., 2017). Beside temperature, many anthropophilic factors play crucial roles in the current success of MBDs (Kilpatrick and Randolph, 2012; Gould et al., 2017; Young, 2018). Human behavior, movement and land use in relation to climate change (population migration, water storage, unplanned urbanization…) are also subjects to explore. Notably, vector control strategies should include a temperature component in testing insecticide efficacy used in public health (Glunt et al., 2018).

Owing to the complex interactions between all partners of the vectorial system, more studies on the role of temperature on viral transmission are required. A better understanding of how transmission cycles interact with changing environments will help to better respond to future arbovirus outbreaks.

## Author Contributions

RB and A-BF wrote the manuscript. Both authors have read and agreed to the published version of the manuscript.

## Conflict of Interest

The authors declare that the research was conducted in the absence of any commercial or financial relationships that could be construed as a potential conflict of interest.

## Footnotes

1 https://www.who.int/news-room/fact-sheets/detail/west-nile-virus
